# Increased nuclear ?-catenin expression in oral potentially malignant lesions: A marker of epithelial dysplasia

**DOI:** 10.4317/medoral.20341

**Published:** 2015-08-04

**Authors:** Montserrat Reyes, Gonzalo Rojas-Alcayaga, Andrea Maturana, Juan-Pablo Aitken, Carolina Rojas, Ana-Verónica Ortega

**Affiliations:** 1 Department of Pathology, Faculty of Dentistry, University of Chile, Santiago, Chile

## Abstract

**Background:**

Deregulation of ?-catenin is associated with malignant transformation; however, its relationship with potentially malignant and malignant oral processes is not fully understood. The aim of this study was to determine and compare the nuclear ?-catenin expression in oral dysplasia and oral squamous cell carcinoma (OSCC).

**Material and Methods:**

Cross sectional study. Immunodetection of ?-catenin was performed on 72 samples, with the following distribution: 21 mild dysplasia, 12 moderate dysplasia, severe dysplasia 3, 36 OSCC including 19 well differentiated, 15 moderately differentiated and 2 poorly differentiated. Through microscopic observation the number of positive cells per 1000 epithelial cells was counted. For the statistical analysis, the Kruskal Wallis test was used.

**Results:**

Nuclear expression of ?-catenin was observed in all samples with severe and moderate dysplasia, with a median of 267.5, in comparison to mild dysplasia whose median was 103.75. Only 10 samples (27.7%) with OSCC showed nuclear expression, with statistically significant differences between groups (*p* < 0.05).

**Conclusions:**

Our results are consistent with most of the reports which show increased presence of ?-catenin in severe and moderate dysplasia compared to mild dysplasia; however the expression of nuclear ?-catenin decreased after starting the invasive neoplastic process. This suggests a role for this protein in the progression of dysplasia and early malignant transformation to OSCC. Immunodetection of ?-catenin could be a possible immune marker in the detection of oral dysplasia.

**Key words:**Oral squamous cell carcinoma (OSCC), ?-catenin, oral dysplasia.

## Introduction

Oral Squamous Cell Carcinoma (OSCC) is the most common malignancy of the oral cavity, and its early diagnosis is essential to improve patient survival and significantly reduce mortality rates (1). Most OSCC may be preceded by visible clinical changes in the oral mucosa; the most common are leukoplakia and erythroplakia (2,3). Both lesions are provisional clinical diagnoses. Therefore, they require a histopathology study for their definitive diagnosis, which can correspond to epithelial hyperplasia, hyperkeratosis, epithelial dysplasia or even carcinoma (4,5). Dysplasia diagnosis is usually quite complex and subjective; consequently some markers of cellular proliferation like Ki-67 have attempted to contribute to a more objective diagnosis (6,7). Some studies have shown that the percentage of Ki-67 positive cells increased according to the degree of severity (8-11). Therefore, this marker is a useful complement of the diagnosis of epithelial dysplasia. However, the molecular alterations and biological mechanisms leading to progression to OSCC are not fully understood and several molecular markers are under study.

The Wnt signaling pathway involves highly conserved genes for proteins whose biological functions are characterized by the growth, proliferation and cellular differentiation (12). Hitherto, we have recognized three different routes serving the Wnt pathway: the canonical pathway or Wnt-?-catenin pathway, the planar cell polarity and the Wnt-dependent Ca2+ (13). The Wnt-?-catenin pathway is regulated by the intracellular levels of ?-catenin protein (14,15), which despites being an effector molecule of the Wnt signal activation, it is also a protein involved in cell adhesion with E-cadherin. In the absence of Wnt signals, ?-catenin is targeted for degradation via the proteasome, by phosphorylation through a multi protein complex composed of a serine/threonine kinase, glycogen synthase kinase 3b (GSK-3b) and a product of tumor suppressor gene: the adenomatous polyposis coli (APC) (15,16). Inhibition of ?-catenin degradation caused by Wnt ligand results in the accumulation of this cytoplasmic protein and subsequent translocation to the nucleus, forming a complex with Lymphoid Stimulating Factor-1/T cell Factor (LEF-1/TCF) that displaces protein Groucho and assumes the function of co-activator, inducing transcription of target genes of Wnt signaling pathway, associated with cell growth and proliferation (17,18). Therefore, if this route presents changes its performance it can lead to uncontrolled cell growth and tumor formation.

The stabilization of ?-catenin is an early event in carcinogenesis. Several reports have demonstrated aberrant activation of Wnt signaling pathway in various human tumors, including colorectal, gastric and melanoma. Given that, there is considerable evidence of abnormalities in the Wnt signaling pathway in tumorigenesis (19,20). Based on the evidence presented, and considering that currently there is no single marker or set of markers that reliably can be used to predict malignant transformation of oral dysplasia, the objective of this study is to determine and compare the nuclear ?-catenin expression in oral dysplasia and OSCC.

## Material and Methods

-1.1 Case selection

The study was approved by the Ethical Committee Board from the Faculty of Dentistry, University of Chile. Informed consent was obtained from all patients according to the Declaration of Helsinki.

The present study examined 72 formalin fixed paraffin-embedded samples from the Pathological Anatomy Laboratory, Faculty of Dentistry, Universidad de Chile: 36 of these samples had a clinical diagnosis of oral leukoplakia or erythroplakia and histopathological diagnosis of dysplasia and 36 had histopathological diagnosis of OSCC. Clinical records were reviewed to collect patient’s demographic data (gender, age) and clinical features of the lesions (clinical diagnosis, localization). Histological classification was assessed independently by two oral pathologists in sections previously stained with hematoxylin-eosin. Dysplasias were classified as: a) mild dysplasia (21 cases) 19 of which (90.47 %) were leukoplakias and 2 (9.5 %) were erythroplakias, b) moderate dysplasia (12 cases), 10 of them (83.3 %) and 2 (16.6 %) were leukoplakias and erythroplakias respectively and severe dysplasia (3 cases). All of them had a clinical diagnosis of leukoplakia. Malignant samples were classified according to their degree of differentiation, corresponding to 19 cases of well differentiated OSCC (52.78 %); 15 cases of moderately differentiated OSCC (41.66 %) and 2 cases of poorly differentiated OSCC (5.55 %). Three normal oral mucosa samples were included as controls.

-1.2 Immunohistochemistry

Four ?m sections from paraffin blocks of each case were obtained and collected in positively charged slides (Lab Cellpath, England). Later they were dewaxed in xylene and rehydrated, descending alcohols to distilled water. Immunohistochemical staining was performed using the streptavidin-avidin-biotin complex (ABC) and the reaction was visualized with diaminobenzidine tetrahydrochloride (DAB). Sections were placed in sodium citrate buffer (pH 6) for 45 minutes in a pressure cooker for antigen retrieval, and then they were washed with PBS for 5 min; endogenous peroxidase activity was blocked by incubating the sections in H2O2 at 3% in methanol at room temperature for 30 minutes. The sections were pre incubated with horse serum for 20 minutes at room temperature and then incubated for 30 minutes with primary antibodies (?-catenin, Dako laboratory, USA and Ki- 67, Cell Marque Laboratory, USA) 1:200 dilution, ready to use (RTU) respectively, in a moist chamber at 37°C. Sections were then washed with PBS for 5 min and incubated with biotinylated secondary antibody for 30 min at 37°C and then with peroxidase-conjugated streptavidin (Universal Detection System Vectastain Elite Kit wide spectrum ABC-HRP, RTU, Vector-USA, EE.UU) for 20 minutes at 37°C. The reaction was finally visualized with diaminobenzidine (DAB) and staining with Harris hematoxylin. Negative controls were obtained by substitution of the primary antibody with PBS.

-1.3 ?-catenin and Ki-67 evaluation

The cellular localization of ?-catenin was classified as membranous, cytoplasmic or nuclear, depending on the immunolocalization pattern. Intensity of membranous staining was assessed as mild, moderate or intense. In non-homogeneous cases, the predominant intensity (over 70% of cells) was recorded. Nuclear and cytoplasmic staining was recorded as positive or negative.

Quantification of nuclear ?-catenin immunostaining was assessed by light microscopic examination with 40x magnification. Five randomly selected fields with presence of dysplastic epithelium and/or neoplastic accordingly were assessed, which were photographed on Olympus BX41 microscope using the SE Premium Micrometrics program. In these photomicrographs, 1000 epithelial cells were counted per case with the Image J program, determining the number of positive cells.

Nuclear distribution of Ki-67 in the epithelium was used to corroborate the degree of dysplasia and architectural changes of the epithelium.

-1.3.1 Statistical Analysis

An exploratory data analysis was performed using descriptive statistics. Kruskal Wallis test was used. A value of significance of 5% or less (*p* < 0.05) to accept statistically significant differences was considered. All statistical tests were performed using Stata 11.0 software.

## Results

-2.1 Patients features

 Table 1 summarizes the clinicopathological features of all cases examined in the present study. The average age for patients with dysplasia was 58 years (range 30 to 79 years), compared with patients with OSCC which was 65 years (range 45 to 91 years).

**Table 1:**
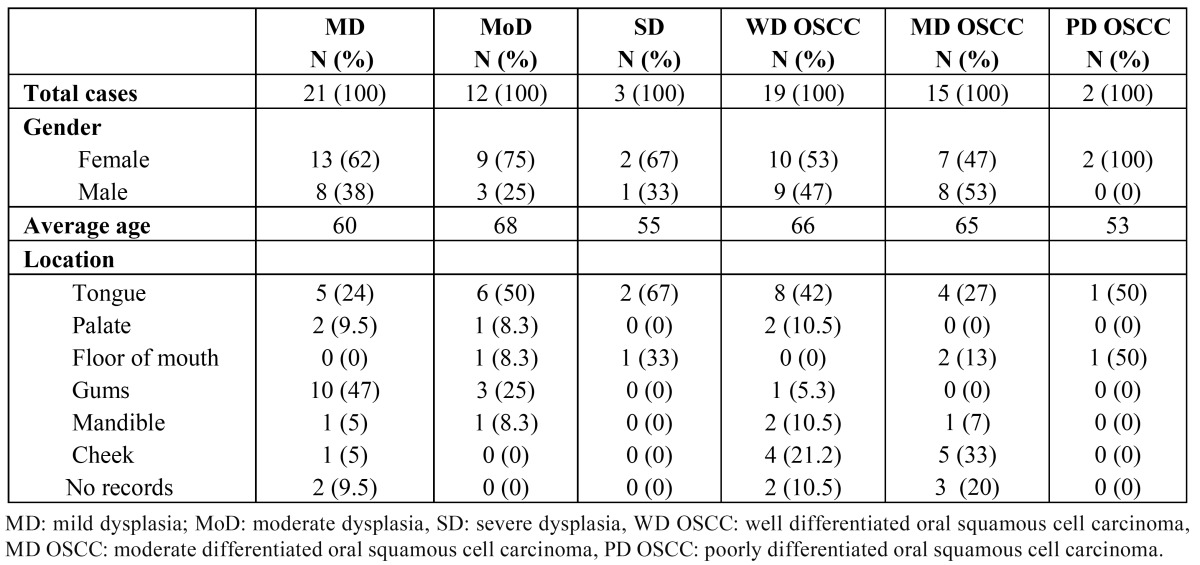
Clinicopathological features of all cases in this study.

The most frequent location in mild dysplasia was in gums, both moderate and severe dysplasia was in tongue and OSCC cases were found mainly in tongue and cheek.

-2.2 Immunohistochemical evaluation of Ki-67

**Figure 1:**
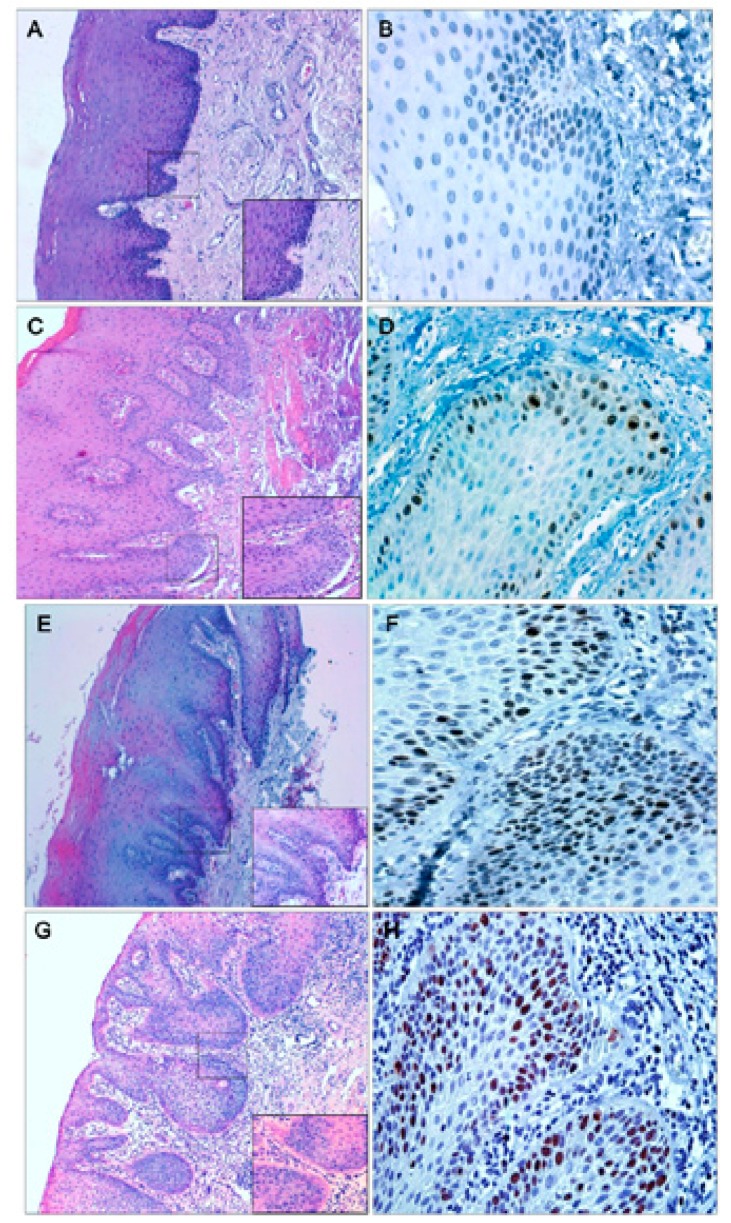
Samples of normal oral mucosa (A), mild dysplasia (C) with hematoxylin-eosin staining. Immunohistochemical localization of Ki-67 at the basal strata of the epithelium in normal oral mucosa (B), in mild dysplasia the protein locates mainly in the lower third of the epithelium (D). Samples of moderate (E) and severe dysplasia (G) with hematoxylin-eosin staining. Immunohistochemical localization of Ki-67 in moderate dysplasia (F) the nuclear expression of Ki-67 is located at the medium and lower third of the epithelium; severe dysplasia (H) immunohistochemical localization of Ki-67 at the lower, medium and higher thirds of the epithelium.

**Figure 2:**
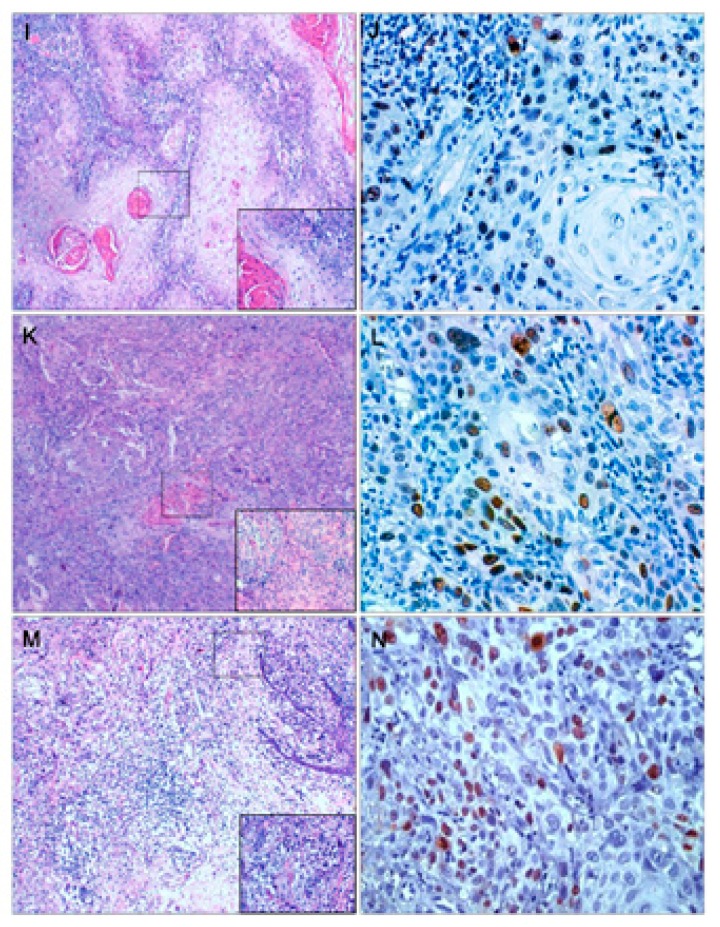
Samples of well differentiated OSCC (I), moderate differentiated OSCC (K) and poorly differentiated OSCC (M) with hematoxylin-eosin staining. Immunolocalization of Ki-67 in well differentiated OSCC (J); moderate differentiated OSCC (L) and poorly differentiated OSCC (N) is observed in all epithelia strata.

Immunostaining with Ki-67 in mild dysplasia was only present in the lower third of the epithelium. In moderate dysplasia, in the middle and lower third of the epithelium, as in severe dysplasia, which was observed in some cases also in the upper third of the epithelium, OSCC samples showed expression of Ki-67 throughout the epithelium. In samples of normal oral mucosa, scarce cell proliferation was observed in the basal layer of the epithelium (Figs. 1a,b,2).

-2.3 Immunohistochemical evaluation of ?-catenin

**Figure 3:**
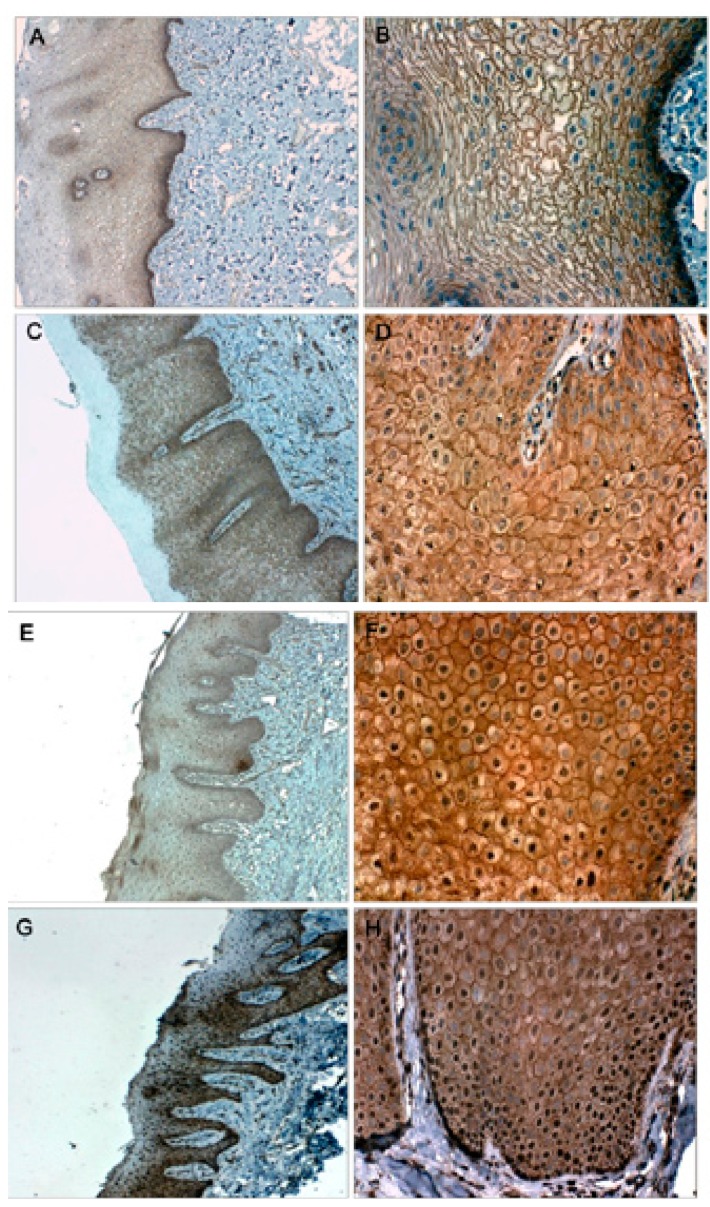
Immunohistochemical localization of ?-catenin in normal oral mucosa (A-B), in which only membrane expression is observed; mild dysplasia (C-D), where the protein is mainly localized at membrane and cytoplasm and the nuclear expression is present but at the lowest degree (D). Immunohistochemical localization of ?-catenin in moderate dysplasia (E-F), where the nuclear expression of ?-catenin is greater; severe dysplasia (G-H) where the protein strongly expressed at the lower layers of the epithelium.

**Figure 4:**
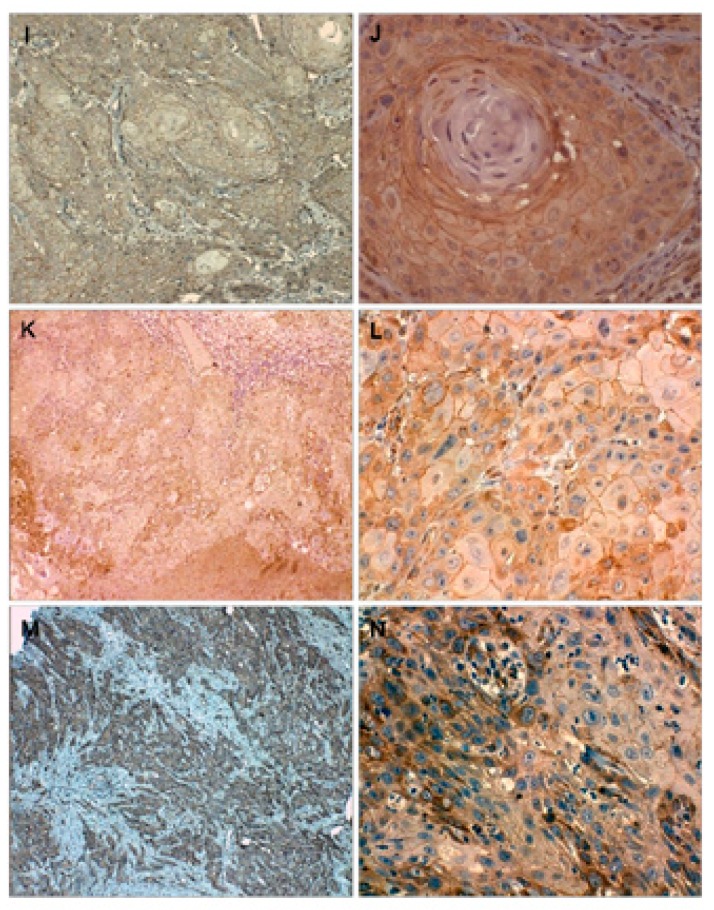
Immunohistochemical localization of ?-catenin in well diferentiated OSCC (I-J), the protein is primarily localized at membranous and cytoplasmic level, the nuclear expression is very low: moderately differentiated OSCC (K-L), the membrane protein expression is lost to a lesser degree of differentiation, as in the poorly differentiated OSCC samples where no nuclear expression of ?-catenin observed (M-N).

In all samples of normal oral mucosa epithelium, localization of ?-catenin was detected in the cell membrane and there was negative immunostaining at cytoplasm and/or nuclear level. All samples of dysplasia and OSCC showed positive cytoplasmic immunostaining in all layers of the epithelium. Expression in the cell membrane in all samples was positive and their intensity decreased to less differentiated and usually lost in the upper layers of the epithelium. In mild dysplasia, 15 of 21 samples (71.42%) showed nuclear labeling, whereas 100% of the samples with moderate dysplasia (12 samples) and severe dysplasia (3 samples) showed nuclear labeling of ?-catenin. Only 10 OSCC samples (27.7%) showed nuclear expression, 6 of them were well differentiated and 4 moderately differentiated ( Table 2). The nuclear expression in moderate and severe dysplasia were mainly located in the lower third of the epithelium, compared to samples from OSCC where in most samples they were located in upper stratas (Figs. 3a,b, 4).

**Table 2:**
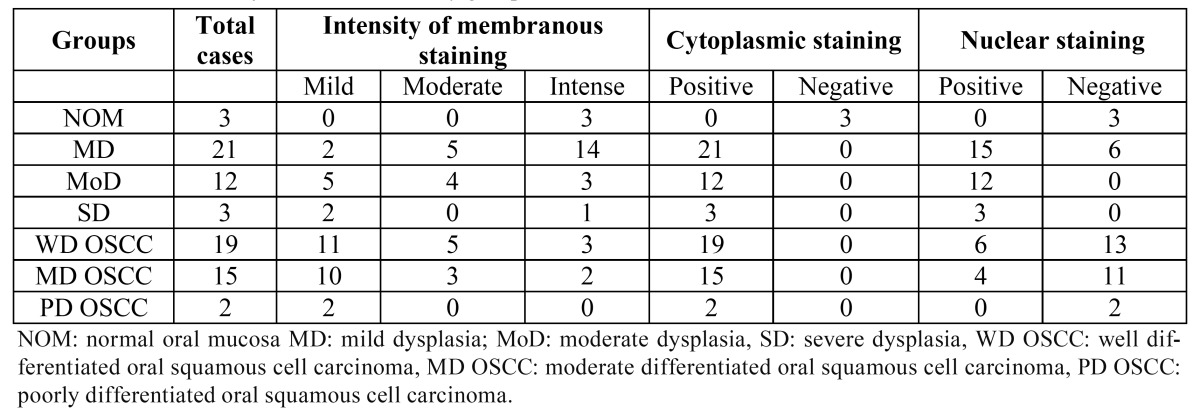
Localization of ?-catenin in the study groups.

Quantification of nuclear expression of ?-catenin in mild dysplasia, moderate dysplasia in conjunction with severe dysplasia and OSCC showed a median of 103.75, 267.5 and 0 respectively. Differences in the nuclear expression of ?-catenin between dysplasia and OSCC were observed (*p*<0.05) ( Table 3).

**Table 3:**
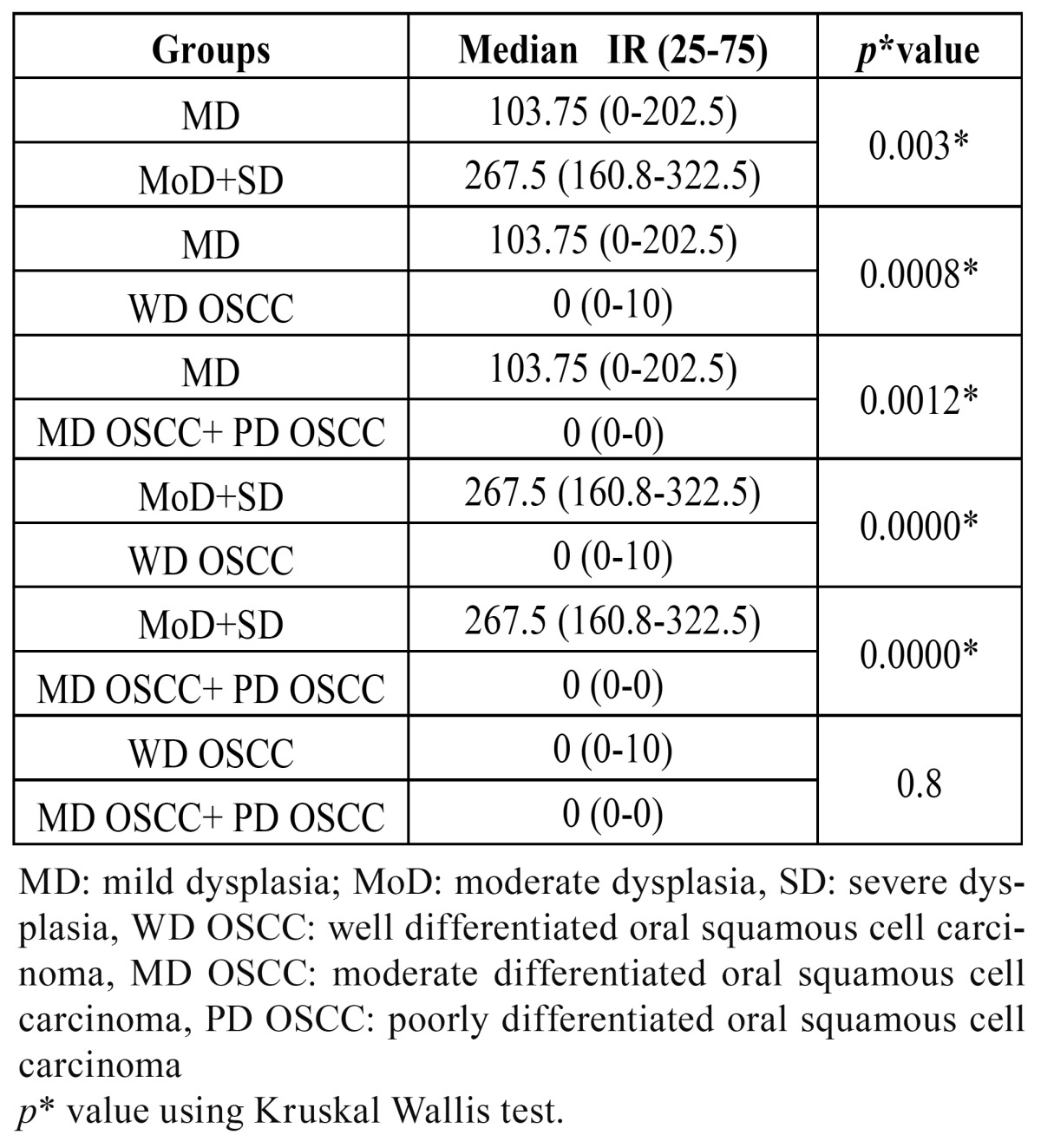
Statistical analyses of differences in nuclear ?-catenin immunostaining in the study groups.

## Discussion

Our objective was to determine and compare the nuclear expression of ?-catenin in oral dysplasia and OSCC. Some studies have been undertaken to try to determine whether the expression of Ki-67 antigen is related to the presence of epithelial dysplasia (21), since the increase in cell proliferation is considered to be feature for malignant progression (22,23). The proliferation marker Ki-67 is expressed in all phases of the cell cycle except G0 (24). Several studies have demonstrated that this protein is a reliable marker of cell proliferation on both malignant and potentially malignant lesions. Zoeller *et al*., (9) studying epithelial dysplasia observed that the percentage of cells expressing Ki-67 increases according to the histopathologic malignancy degree. Saito *et al*., (25) found that expression of Ki-67 increased according to cell proliferation. Moreover, Mimica *et al*., (26) and Cambruzzi *et al*., (27) showed an increase in Ki-67 to low degree of epithelial differentiation in OSCC. Therefore, as these authors suggest, Ki-67 is a good marker of cell proliferation and it also makes it possible to establish the dysplasia degree with more certainty. Given that, this marker was used in this study to confirm the degree of epithelial dysplasia on samples previously diagnosed with hematoxylin-eosin staining, confirming that cell proliferation increased with greater severity of injuries, corroborating the diagnosis and the severity of each of the dysplasias included in our study.

The critical role in Wnt/?-catenin signaling pathway in the development of many human tumors prompted us to investigate if nuclear expression of ?-catenin in dysplasia and OSCC exists, since this molecule is not only an essential integral part in cell adhesion (28), but it also plays an important role in cell proliferation induction. Cytoplasmic accumulation and subsequent nuclear translocation of ?-catenin may be the result of canonical Wnt signaling pathway activation or to pathway impairment due to mutations in some of its components and therefore its cytoplasmic overexpression as its nuclear localization is known to be associated with malignant transformation in different types of cancers (29-31). However, its expression in oral dysplasia and OSCC has been poorly investigated. A considerable number of ?-catenin studies in this type of oral lesions focuses on its expression as a component of the cell adhesion system, therefore to their membrane detection, since it has been found a relationship between ?-catenin expression and cell differentiation degree (32), where the decrease in membrane expression of this protein has been associated with loss of cell differentiation (33). This is evidenced by the reduced membrane expression of this protein (32). Williams *et al*., (34) showed that membranous expression of ?-catenin was reduced both in severe dysplasia and carcinoma in situ. Bankfalvi *et al*., (35,36) indicate that investigated areas of dysplastic mucosa adjacent to tumor are associated with a limited loss of ?-catenin. Laxmidevi *et al*., (33) describe that membranous localization of ?-catenin was correlated with the degree of differentiation in OSCC, showing a decrease of it to less differentiated OSCC, just as the results obtained by Yu *et al*., (37) Gasparoni *et al*., (38) and Bagutti *et al*., (39). Therefore, the reduced membrane expression and increased cytoplasmic expression of ?-catenin reflect the aggressive nature of OSCC. Our results agree that a lesser differentiation degree in OSCC is associated with a progressive reduction of membranous expression of ?-catenin. However, there are few studies that indicate the expression of ?-catenin in human oral dysplasia and most of them focus on OSCC.

As for ?-catenin nuclear expression some authors point out that there is an intracytoplasmic and nuclear accumulation of ?-catenin in 62.5% of dysplastic lesions induced in oral mucosa of rats (40). Ishida *et al*., (41) indicates that ?-catenin nuclear expression increased during the severity progression of dysplastic oral leukoplakia, also observing nuclear expression in 10 out of 15 samples from OSCC and reduced membrane expression of the protein which they associated with the appearance of cell invasion or metastasis. Our results show an increase nuclear expression of ?-catenin to a greater severity of oral dysplasia, but not in OSCC, in where nuclear expression was much lower compared to the aforementioned study, in which they only indicate the presence of the protein and not the amount of it present in a lesion, or the location of the protein in the epithelial layer. This is relevant because it indicates that ?-catenin nuclear expression on basal cells of the lower strata of the epithelium will show that this protein might function as a transcription factor for proliferative activation of these cells, which agrees on our results, where the nuclear localization of ?-catenin was mainly found on lower and middle epithelial layers as the severity of dysplasia was greater.

Our results are in line with those reported in previous studies in which increased presence of ?-catenin is shown in moderate and severe dysplasia compared to oral mucosa mild dysplasia. The results of this study suggest that nuclear expression of this protein in epithelia lower strata may be related to cell proliferation, and more importantly that most of the nuclear expression of ?-catenin in moderate and severe dysplastic lesions would be involved in the malignant transformation towards the development of OSCC, which is why it is suggested that ?-catenin is a potential marker for dysplastic lesions of the oral cavity.
